# Identification of Acid Hydrolysis Metabolites of the Pimelea Toxin Simplexin for Targeted UPLC-MS/MS Analysis

**DOI:** 10.3390/toxins15090551

**Published:** 2023-09-05

**Authors:** Zhi Hung Loh, Natasha L. Hungerford, Diane Ouwerkerk, Athol V. Klieve, Mary T. Fletcher

**Affiliations:** 1 Queensland Alliance for Agriculture and Food Innovation (QAAFI), The University of Queensland, Health and Food Sciences Precinct, Coopers Plains, QLD 4108, Australia; zhihung.loh@uq.edu.au (Z.H.L.); n.hungerford@uq.edu.au (N.L.H.); diane.ouwerkerk@daf.qld.gov.au (D.O.); a.klieve@uq.edu.au (A.V.K.); 2 Agri-Science Queensland, Department of Agriculture and Fisheries (DAF), Ecosciences Precinct, Dutton Park, QLD 4102, Australia

**Keywords:** Pimelea, simplexin, plant toxin, mass spectrometry, metabolite, rumen, fermentation, acid hydrolysis

## Abstract

Pimelea poisoning of cattle is a unique Australian toxic condition caused by the daphnane orthoester simplexin present in native Pimelea pasture plants. Rumen microorganisms have been proposed to metabolise simplexin by enzymatic reactions, likely at the orthoester and epoxide moieties of simplexin, but a metabolic pathway has not been confirmed. This study aimed to investigate this metabolic pathway through the analysis of putative simplexin metabolites. Purified simplexin was hydrolysed with aqueous hydrochloric acid and sulfuric acid to produce target metabolites for UPLC-MS/MS analysis of fermentation fluid samples, bacterial isolate samples, and other biological samples. UPLC-MS/MS analysis identified predicted hydrolysed products from both acid hydrolysis procedures with MS breakdown of these putative products sharing high-resolution accurate mass (HRAM) fragmentation ions with simplexin. However, targeted UPLC-MS/MS analysis of the biological samples failed to detect the H_2_SO_4_ degradation products, suggesting that the rumen microorganisms were unable to produce similar simplexin degradation products at detectable levels, or that metabolites, once formed, were further metabolised. Overall, in vitro acid hydrolysis was able to hydrolyse simplexin at the orthoester and epoxide functionalities, but targeted UPLC-MS/MS analysis of biological samples did not detect any of the identified simplexin hydrolysis products.

## 1. Introduction

**Figure 1:**
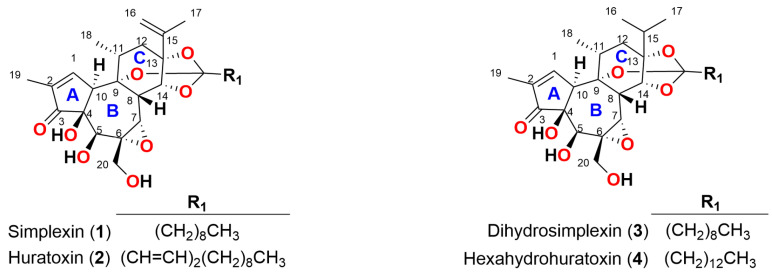
Structure of simplexin (**1**) showing the daphnane skeleton structure and C_9_ saturated fatty acid chain attached by an orthoester linkage. Huratoxin (**2**) by comparison contains a C_13_ tri-unsaturated fatty acid chain ((1*E*, 3*E*)-trideca-1,3-dienyl) attached by an orthoester linkage, together with the dihydro and hexahydro forms of these orthoesters **3** and **4**. Daphnane orthoesters have a tricyclic 5/7/6-membered ring system labelled ring A, B and C respectively.

Pimelea poisoning of cattle is a unique Australian poisoning syndrome caused by the daphnane orthoester simplexin ((**1**), Figure 1) present in native Pimelea pasture plants, particularly *Pimelea trichostachya*, *P. simplex*, and *P. elongata*, which occur widely over much of Australia’s inland grazing regions [1]. The Australian beef industry has been reported to suffer estimated losses of up to AUD $50 million annually due to production losses and additional management work performed to combat Pimelea poisoning [1]. Cattle are particularly sensitive to simplexin, which, as a potent activator of protein kinase C (PKC), causes constriction of bovine pulmonary venules, right ventricular dilation, and circulatory failure with subsequent oedema and anaemia from hypervolemia [2,3,4]. It is also a severe irritant, causing chronic diarrhoea [5]. The C-4, C-5, and C-20 hydroxyl groups of simplexin are proposed to be important for PKC binding [6,7]. The related daphnane orthoester huratoxin (**2**) is also found in Pimelea plant material [8] and presumably exerts similar toxicity, as huratoxin and simplexin only differ in the alkyl chain of the orthoester (Figure 1).

A previous cattle feeding trial with increasing low doses of simplexin demonstrated that cattle were able to develop some resistance against the effects of the toxin over time, which suggested simplexin degradation had occurred potentially through adaptation of the rumen microbiome [9]. Consistent with this hypothesis, simplexin was not detected in any of the blood or other animal tissues collected during this previous trial [9]. Phorbol esters, which have a similar diterpene skeleton to simplexin (and are also PKC activators), have been reported to be degraded during microbial fermentation [10,11,12]. This degradation was argued to be due to enzymatic hydrolysis of phorbol esters during the fermentation process. Rumen microorganisms are known to have the ability to metabolise and detoxify a wide range of plant toxins with the demonstrated potential to improve ruminant health and welfare [13]. Likewise, ruminal hydrolysis of simplexin has the potential to provide a much-desired tool in mitigating impacts of Pimelea poisoning by degrading the toxin before it is absorbed from the digestive tract. The aim of the current study was thus to investigate ruminal hydrolysis of simplexin and associated metabolic pathways through the analysis of putative simplexin hydrolysis metabolites.

**Figure 2:**
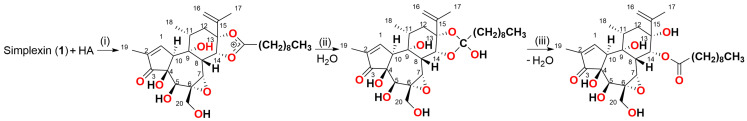
Proposed mechanism for acid (HA) mediated three-step orthoester hydrolysis mechanism of simplexin (**1**) where step (i) is the formation of the carboxonium ion, step (ii) is the addition of the nucleophile H_2_O and step (iii) is the decomposition of the hemiortho ester.

**Figure 3:**
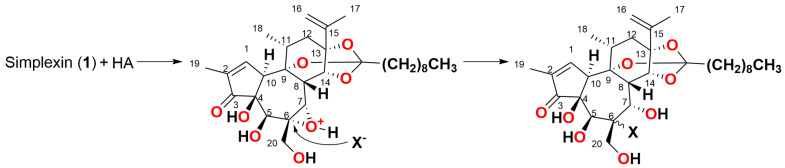
Proposed mechanism for acid (HA) mediated epoxide hydrolysis mechanism of simplexin (**1**) where X is either water or an anion such as chloride.

One of the postulated simplexin degradation/deactivation pathways is the hydrolysis of the simplexin orthoester moiety (Figure 2), which would alter the hydrophobicity and hence the metabolic fate of this toxin. Orthoester hydrolysis to the corresponding ester is proposed to be a three-step mechanism that involves the formation of the carboxonium ion, followed by the addition of a nucleophile (water) and the decomposition of the hemi-orthoester producing an ester and alcohol product [14,15]. Further hydrolysis of the remaining ester would ultimately produce a triol in the case of simplexin orthoester hydrolysis. However, a certain reluctance of the cage-like orthoester of the daphnane skeleton C ring structure to undergo hydrolysis has been noted in other daphnane orthoesters, and this is attributed to the conformational change to the cyclohexane ring, which is forced to adopt the usually less favoured boat form due to the presence of the orthoester cage [14,16,17]. An alternate proposed simplexin degradation pathway is the ring-opening of the simplexin 6,7-epoxide (Figure 3), which could potentially result in the conformational changes in simplexin that could alter its bioactivity. Hydrolysis of the epoxide of the related compounds dihydrosimplexin (**3**) and hexahydrohuratoxin (**4**) (Figure 1) have previously been achieved under acidic conditions [8,17].

The rumen is home to anaerobic microorganisms that undertake the anaerobic fermentation of ingested feed materials, producing volatile fatty acids that are the primary energy source for cattle [18]. These microorganisms produce enzymes that hydrolyse food materials into metabolites able to be absorbed by the bovine blood and/or lymphatic systems. Some enzymes possess a catalytic triad in the active site (e.g., Ser-His-Asp/Glu in lipases and serine proteases) that are involved in hydrolysis reactions and that result in the overall nucleophilic attack on substrates [19]. It is proposed that a candidate bacterium in Pimelea resistant ruminants may be able to secrete an enzyme with such a catalytic triad with the capacity to hydrolyse simplexin, resulting in the opening of the orthoester and/or the epoxide. However, it is first necessary to identify potential simplexin hydrolysis products.

The current study sought to investigate the hydrolysis of simplexin with strong acids (hydrochloric acid (HCl) and sulfuric acid (H_2_SO_4_)) with the intention of producing potential degradation metabolites and providing target metabolites for ultra performance liquid chromatography tandem mass spectrometry (UPLC-MS/MS) analysis of fermentation fluid samples, bacterial isolate samples, and other biological samples. Hydrolysed products identified in the biological samples could also be used to select for simplexin degrading bacteria from fermentation fluids and microbial isolate samples, and to detect potential simplexin metabolites in blood samples from cattle consuming Pimelea.

## 2. Results and Discussion

### 2.1. HRAM Analysis of Simplexin and MS Fragments

**Figure 4:**
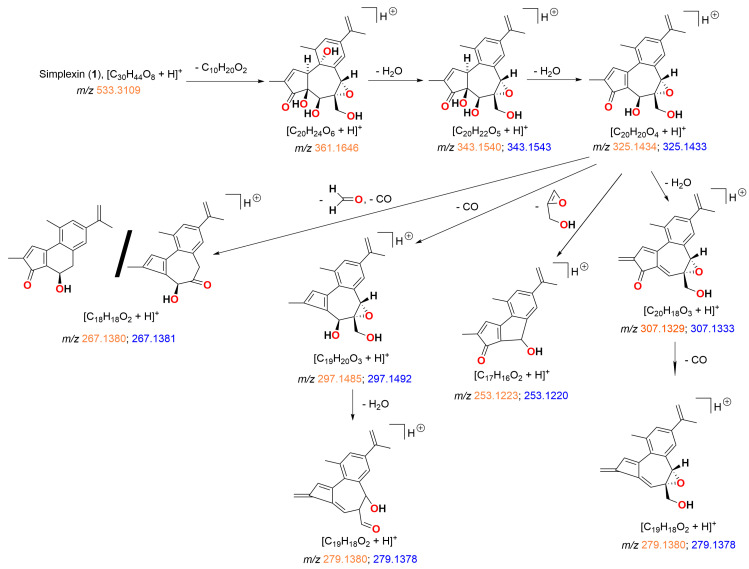
Proposed fragmentation pathway leading to some characteristic fragment ions of protonated simplexin (**1**). HRAM analysis of fragment ions (*m*/*z*) shown as calculated (orange) and measured (blue) confirmed the molecular composition of each ion.

Analysis of purified simplexin utilising a Thermo Scientific Q-Exactive Orbitrap HRAM mass spectrometer (Thermo Fisher Scientific, Waltham, MA, USA) enabled the confirmation of molecular formula for both simplexin and postulated fragment ions (Figure 4). LC-MS/MS methods have previously been developed for the analysis of simplexin in plant material using a triple quadrupole spectrometer [1]. The Q-Exactive hybrid quadrupole-Orbitrap mass spectrometer was used in the current study due to its accurate mass measurements and high resolving power at MS/MS levels, which allows identification and quantification of molecules and their metabolites at parts per billion concentrations [20].

Our postulated structures of fragmentation ions for simplexin (**1**) were based on fragment ion structures fragmentation pathway of protonated huratoxin (**2**) as proposed by Trinel et al. [21]. Our measured accurate mass for molecular and fragment ions was in good agreement and provided confirmation of the molecular composition for the proposed MS fragment ions shown in Table 1 and Figure 4. The ion fragment *m*/*z* 361 corresponds to the basic daphnane core and originates from the neutral loss of the acyl chain from simplexin. This is the same diagnostic ion called “huratoxigenin” by Trinel et al. [21] in reference to its formation from huratoxin (**2**). The mechanism of acyl loss was proposed as a simultaneous double six-membered McLafferty-type rearrangement with loss of protons H-8 and H-12 and remote hydrogen rearrangement on OH at C-9 [21]. Sequential dehydrations of the ion fragment *m*/*z* 361 at C-9, C-4, and C-5 then provides *m*/*z* 343, 325, and 307 ions (Figure 4), respectively, with loss of CO at C-3 and ring contraction providing further products. Notably, the base peak *m*/*z* 253 is formed by double dehydration (−36 amu) to *m*/*z* 325 and contraction of the seven-membered ring leading to a neutral loss of a fragment containing the C-6/C-7 epoxide and the C-20 hydroxymethylene (Figure 4).

**Figure 5:**
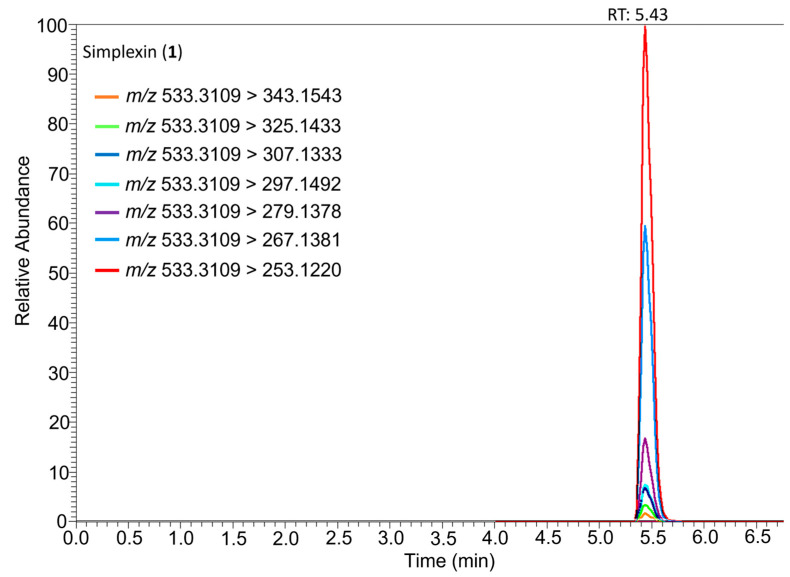
Ion chromatogram showing predominant fragment transitions of protonated simplexin (**1**) in positive ionisation mode.

The ion chromatograms of the seven most predominant fragment transitions of protonated simplexin (**1**) are well superimposed at the same retention time as shown in Figure 5. The major fragment ions arising from triple quadrupole LC-MS/MS analysis have previously been reported [1], but molecular composition of fragment ions was not then available. The HRAM analysis (Table 1) reported in this study is consistent with that reported by Chow et al. [1] and confirms, for the first time, the molecular composition of each fragment ion as shown in Figure 4. This knowledge is then useful in predicting expected ions from hydrolysis experiments.

### 2.2. HCl Mediated Hydrolysis of Simplexin

**Figure 6:**
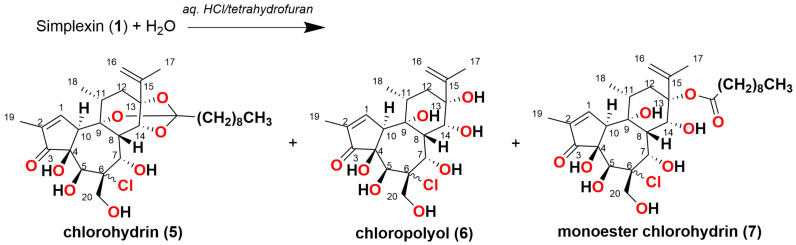
Proposed structure of HCl hydrolysis products from predicted molecular formulae for chlorohydrin (**5**), chloropolyol (**6**) and monoester chlorohydrin (**7**). Stereochemistry of the B ring and regiochemistry were not determined in hydrolysis products **5**, **6** and **7**.

**Figure 7:**
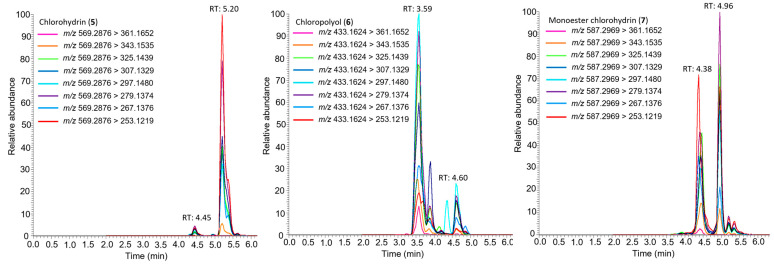
Extracted ion chromatograms of chlorohydrin (**5**), chloropolyol (**6**) and monoester chlorohydrin (**7**) showing its transitions in positive ionisation using typical simplexin fragment ions.

**Figure 8:**
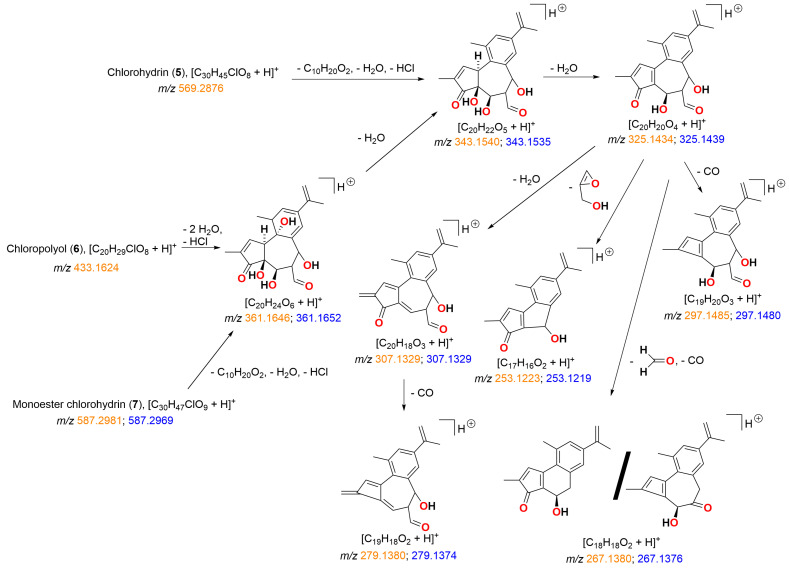
Proposed fragmentation pathway and fragment ion structure for HCl hydrolysis products **5**, **6** and **7**. HRAM analysis of fragment ions (*m*/*z*) shown as calculated (orange) and measured (blue) confirmed the molecular composition of each ion.

HCl mediated hydrolysis of simplexin provided three sub-milligram products that were tentatively identified by mass spectral interpretation (but not isolated) and tentatively named chlorohydrin (**5**), chloropolyol (**6**), and monoester chlorohydrin (**7**), respectively. These predicted products (Figure 6) were based on predicted molecular formulae of C_30_H_45_ClO_8_, C_20_H_29_ClO_8_, and C_30_H_47_ClO_9_ (Table 2), which corresponded to the extracted ion chromatograms shown in Figure 7. The isotope ^35^Cl/^37^Cl pattern of the molecular formula of hydrolysis products **5**, **6**, and **7** also matched with the calculated molecular formula of the respective products (Figure S1). The hydrolysis products **5**, **6**, and **7** also showed both major and minor peaks with similar fragmentation ions in the mass spectra (Figure 7), which are presumed to be regioisomers or stereoisomers, with the dominant isomers presumed to be the C-6 chloride as depicted in Figure 6 based on the NMR evidence provided by Sakata et al. [17] for the dihydro-chloropolyol derived from the corresponding epoxide in hexahydrohuratoxin. Sakata et al. [17] stated: “the entering group was apparently located on C-6 and the hydroxyl group from the epoxide on C-7”. Freeman et al. [8] also reported the same dihydro-polyol from a 1:1 mixture of dihydrosimplexin (**3**) and hexahydrohuratoxin (**4**), although these authors mistakenly depicted chemical structures with the chloride at C-7. The 6-chloro structures (Figure 6) are consistent with our proposed MS fragmentation scheme for these chloro-metabolites **5**, **6**, and **7** (Figure 8).

The mass spectra of all hydrolysis products **5**, **6**, and **7** showed strong intensity peaks of [M + H]^+^ adduct in positive ionisation mode similar to [M + H]^+^ adduct formed in simplexin. Products **5**, **6**, and **7** showed similar fragment ions *m*/*z* 361, 343, 325, 307, 297, 279, 267, and 253 (Figure 7 and Figure 8) to simplexin (Table 1, Figure 4 and Figure 5). The structures of these fragments *m*/*z* 361, 343, 325, 307, and 297 in the hydrolysis products were proposed to have alternative structures with differing oxidation at C-6 and C-20 position of ring B (Figure 8) when compared to those described above for simplexin MS fragmentation (Figure 4) due to the presence of the presence of the C-6 chloride in **5**, **6**, and **7** and the elimination of HCl in the formation of these fragments. Fragment *m*/*z* 279 arises from the loss of CO from fragment *m*/*z* 307. Both fragments *m*/*z* 267 and 253 were formed as observed for simplexin, as described in Figure 4. The proposed alternative ion structures are one of the many possibilities that can be formed during MS fragmentation as fragment ions of the same mass can form different structures by different pathways depending on the parent compound and the ion ratio pattern produced. However, further analysis is required to determine the regiochemistry and stereochemistry of the predicted hydrolysis products as the relative intensities of the shared fragment ions differ between the hydrolysis products and simplexin.

HRAM analysis identified a [M + H]^+^ ion *m*/*z* 569.2870 that corresponded to the molecular formula C_30_H_45_ClO_8_ (calculated [M + H]^+^ *m*/*z* 569.2876) and was predicted to be the chlorohydrin (**5**) derivative of simplexin (Figure 6). Fragment *m*/*z* 569 > 253 also showed high relative intensity as observed in simplexin fragmentation to provide a *m*/*z* 253 fragment ion (Table 1 and Figure 5). Chlorohydrin (**5**) was predicted to have formed from opening of the 6,7-epoxide group at the B-ring via an intermediate carbocation with the chloride ion attached on the C-6 position of ring B, regiochemistry, which is in agreement with Sakata et al. [17].

The second hydrolysis product was identified by HRAM analysis and predicted to have molecular formula C_20_H_29_ClO_8_ with [M + H]^+^ ion *m*/*z* 433.1614 (calculated [M + H]^+^ *m*/*z* 433.1624). This second product was predicted to be the chloropolyol ((**6**), Figure 6) from cleavage of the orthoester moiety in chlorohydrin (**5**). A further hydrolysis product was identified by HRAM analysis with [M + H]^+^ ion *m*/*z* 587.3003 corresponding to the molecular formula C_30_H_47_ClO_9_ (calculated [M + H]^+^ *m*/*z* 587.2982). The proposed hydrolysis product was given the simple name monoester chlorohydrin ((**7**), Figure 6). The structure of the product was predicted to be a hexahydroxyl compound with its orthoester opened without the complete cleavage of the ester side chain linkage. Formation of the monoester chlorohydrin (**7**) in the current study is consistent with the mechanism of orthoester hydrolysis where an orthoester in the presence of acid would form a carboxonium ion to which addition of a nucleophile (water) leads to the decomposition of the hemiorthoester producing an ester and alcohol products [14].

It is thus proposed that simplexin hydrolysis with aqueous hydrochloric acid yielded the chlorohydrin (**5**) as a result of ring-opening of the epoxide. Subsequent reaction then yielded the chloropolyol (**6**) and monoester chlorohydrin (**7**) as a result of complete and partial orthoester hydrolysis, respectively.

### 2.3. H_2_SO_4_ Mediated Hydrolysis of Simplexin

**Figure 9:**
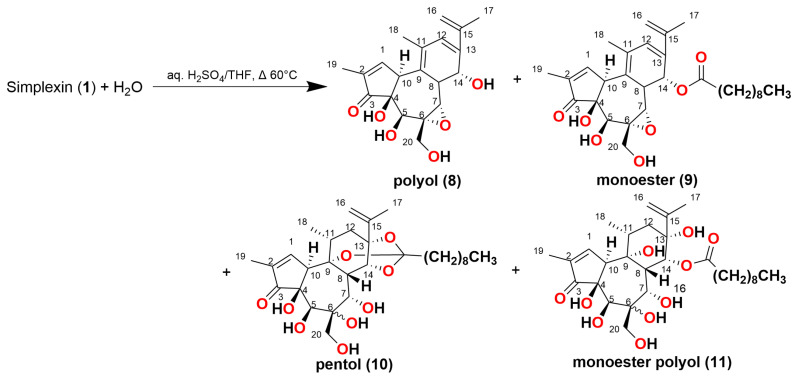
Proposed structure of the H_2_SO_4_ mediated hydrolysis products from predicted molecular formulae for polyol (**8**), monoester (**9**), pentol (**10**) and monoester polyol (**11**), with stereochemistry of the B ring and regiochemistry not determined.

**Figure 10:**
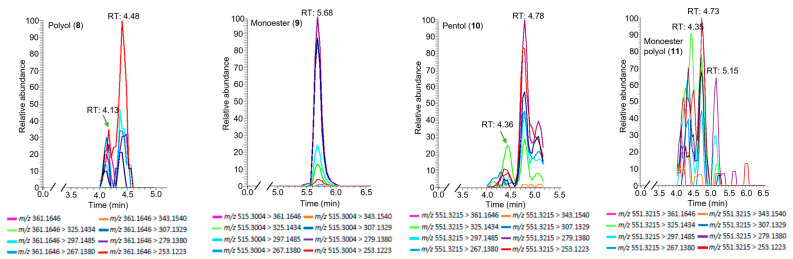
Extracted ion chromatogram of polyol (**8**), monoester (**9**), pentol (**10**) and monoester polyol (**11**) showing its transitions in positive ionisation mode using typical simplexin fragment ions.

In order to avoid chlorinated products, sulfuric acid was then employed as the acid catalyst in the hydrolysis of simplexin (Figure 9). Four products were identified based on spectral fragmentation and HRAM data corresponding to molecular formulae C_20_H_24_O_6_, C_30_H_42_O_7_, C_30_H_46_O_9_, and C_30_H_48_O_10_ and were given the trivial names polyol (**8**), monoester (**9**), pentol (**10**), and monoester polyol (**11**) (Figure 9 and Figure 10). For polyol (**8**), pentol (**10**), and monoester polyol (**11**), there were both major and minor peaks with similar fragmentation ions (Figure 10) but different ion ratios in the spectra which are again presumed to be regioisomers or stereoisomers.

**Figure 11:**
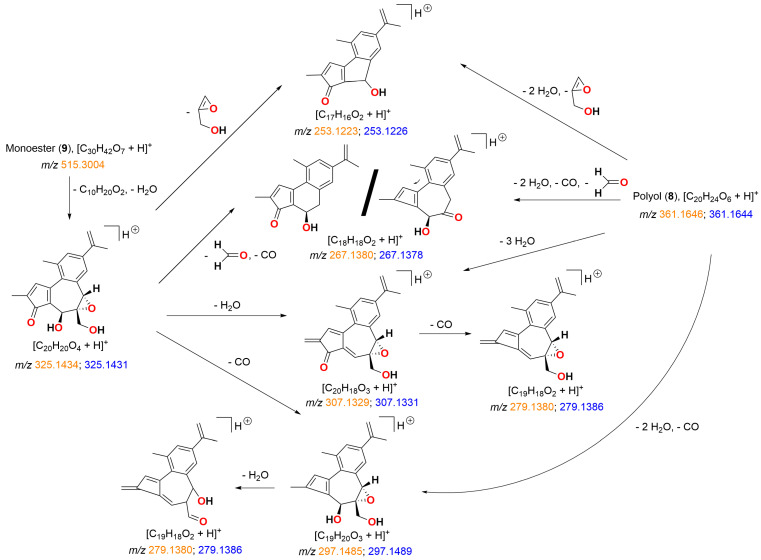
Proposed fragmentation pathways and fragment ion structures for H_2_SO_4_ hydrolysis products polyol (**8**) and monoester (**9**). HRAM analysis of fragment ions (*m*/*z*) shown as calculated (orange) and measured (blue) confirmed the molecular composition of each ion.

**Figure 12:**
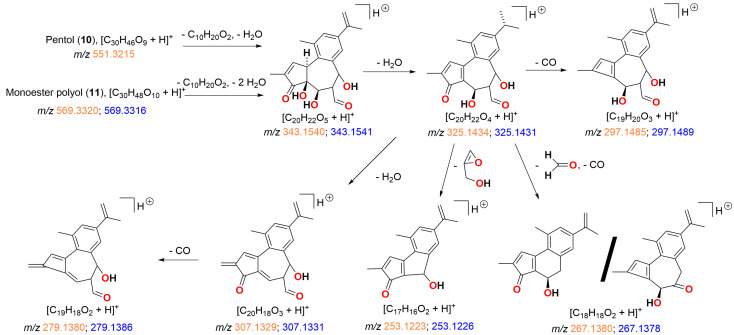
Proposed fragmentation pathways and fragment ion structures for H_2_SO_4_ hydrolysis products pentol (**10**) and monoester polyol (**11**). HRAM analysis of fragment ions (*m*/*z*) shown as calculated (orange) and measured (blue) confirmed the molecular composition of each ion.

The H_2_SO_4_ hydrolysis products showed preference to form the molecular ion proton adduct [M + H]^+^ in positive ionisation mode similar to simplexin (**1**). Products **8**, **9**, **10**, and **11** shared similar fragment ions to simplexin (**1**) (Table 1) and to HCl hydrolysis products **5**, **6**, and **7** (Figure 7). Fragment ions *m*/*z* 343, 325, 307, 297, 279, 267, and 253 in polyol (**8**) and monoester (**9**) (Figure 11) were proposed to have similar structures and derivation to the proposed fragment ions from simplexin fragmentation (Figure 4). On the other hand, fragments *m*/*z* 343, 325, 307, and 297 of pentol (**10**) and monoester polyol (**11**) (Figure 12) were proposed to be similar to the proposed fragment ions of simplexin but differ at position C-20 of the structure with the additional loss of a water molecule from the hydrolysed 6,7 epoxide in both **10** and **11**. However, there are multiple structures could also be formed from ions of the same mass by different fragmentation pathways. The further fragment *m*/*z* 279 was proposed to be a loss of CO from fragment *m*/*z* 307, while fragments *m*/*z* 267 and 253 were proposed to undergo the same proposed fragmentation pathway as simplexin (Figure 4). Differences in the relative intensities of the shared fragment ions between these hydrolysis products (Figure 10) and simplexin (Figure 5) likely reflects changes in the daphnane skeleton structure. The regiochemistry and stereochemistry of the predicted products (Figure 10) have not been determined.

The molecular formula C_20_H_24_O_6_ identified by HRAM analysis with [M + H]^+^ ion *m*/*z* 361.1644 (calculated [M + H]^+^ *m*/*z* 361.1646) was proposed to be polyol (**8**), which is a polyol derivative of simplexin with epoxide linkage still intact (Figure 9). The simplexin derivative is thought to undergo complete hydrolysis of the orthoester group with elimination of H_2_O and the carboxylic acid from the simplexin skeleton. The product with molecular formula C_30_H_42_O_7_ was proposed to be monoester (**9**) of simplexin, which was identified by HRAM analysis with [M + H]^+^ ion *m*/*z* 515.3011 (calculated [M + H]^+^ *m*/*z* 515.3004). Its structure is predicted to have its orthoester partially hydrolysed to a monoester (Figure 9) and two water molecules are also eliminated.

The product with molecular formula of C_30_H_46_O_9_ was predicted to be the pentol (**10**) derived from simplexin with HRAM analysis identifying the [M + H]^+^ ion to be *m*/*z* 551.3220 (calculated [M + H]^+^ *m*/*z* 551.3215). The structure was predicted to be pentol (**10**) with its 6,7-epoxide side chain opened, forming overall a pentahydroxyl structure due to nucleophilic attack of water (Figure 9). This compound is the hydroxyl analog of chlorohydrin (**5**). In the absence of the chloride ion from HCl hydrolysis, water adds to the carbocation intermediate following acid catalysed ring opening. The proposed structure of pentol (**10**) in the current study is in agreement with the corresponding dihydro-pentol reported from sulfuric acid hydrolysis of hexahydrohuratoxin (**4**), in which the epoxide analogously was opened, as confirmed by NMR [17]. Sulfuric acid hydrolysis reaction of hexahydrohuratoxin (**4**) by Sakata et al. [17] also reported a small amount of methoxyhydrin resulting from reaction with the methanol solvent. Such methoxy products were not observed in the present study due to our use of tetrahydrofuran, rather than methanol. The molecular formula C_30_H_48_O_10_ for the final hydrolysis product suggested a product of structure monoester polyol (**11**). HRAM analysis identified the [M + H]^+^ ion to be *m*/*z* 569.3316 (calculated [M + H]^+^ *m*/*z* 569.3320). The structure was proposed to be a heptol with its orthoester partially hydrolysed to the ester (Figure 9). Monoester polyol (**11**) shows the presence of at least three isomers (Figure 10) which are most likely the positional isomers with the remaining ester linkage at each of the orthoester hydroxyl position C-9, C-13, and C-14. It is thus proposed that simplexin hydrolysis with sulfuric acid yielded products due to acid catalysed ring-opening of the epoxide with water, as well as acid-catalysed partial or complete opening of the orthoester bonds and elimination of water from the daphnane skeleton. The harsh conditions required for hydrolysis of this cage-like orthoester are ascribed to the boat conformation of the cyclohexyl C-ring (Figure 1) and the stereoelectronic/non-bonded interactions in the acid hydrolysis intermediates in which this ring adopts a skew-boat conformation [17].

### 2.4. Analysis of Biological Samples for Acid Hydrolysed (H_2_SO_4_) Simplexin Products

The previous cattle Pimelea feeding trial [9] with increasing low doses of simplexin suggested that the cattle rumen flora had adapted over time and that simplexin degradation had potentially occurred in the rumen. As a follow-on study, rumen fluid from these trial cattle and other field collected rumen samples were used in the present study as starters for a series of in vitro fermentation studies using a benchtop fermentation system fed daily with milled Pimelea plant material and with daily sampling of fermentation fluids for up to 56 days [22]. Then, bacterial isolates obtained from these fermenter studies were incubated in selective culture media containing semi-pure simplexin and tested for their ability to degrade simplexin. All fermentation fluid samples and bacterial isolate samples were subjected to targeted UPLC-MS/MS analysis to search for the same H_2_SO_4_ simplexin hydrolysed products **8**, **9**, **10**, and **11**, as shown in Figure 9. This targeted analysis utilised the molecular ions identified in Table 2 for compounds **8**, **9**, **10**, and **11** and the fragment ions *m*/*z* 343, 325, 307, 297, 279, 267 and 253. The HCl hydrolysis products **5**, **6**, and **7** were not targeted as the products are not likely to be formed during metabolism due to the low abundance of chloride ions in the rumen. It was hoped that the H_2_SO_4_ hydrolysis products would match the products formed from simplexin enzymatic hydrolysis. However, none of the targeted H_2_SO_4_ hydrolysis products **8**, **9**, **10**, and **11** were detected in any of the in vitro study samples.

Simplexin quantification was also performed on the fermentation fluid samples and bacterial isolate samples using targeted UPLC-MS/MS analysis. Despite the absence of products **8**, **9**, **10**, and **11** in the daily fermentation fluid samples, the simplexin concentration was observed to decrease over time in the rumen-based in vitro fermentation trials [22]. However, the negative control incubation containing sterilised (autoclaved) rumen fluid and milled Pimelea plant material also showed a similar decrease in simplexin concentration. The adhesion of hydrophobic simplexin to the rumen bacteria cell membranes or other organic (plant) matter during these fermenter incubations was seen as a possible explanation for this apparent decrease in simplexin level in the negative control. Nonetheless, it was determined that fermentations started with ingesta fluids from cattle, goat, kangaroo, and sheep had significantly lower simplexin levels than seen in the negative control at the same time points and may have indicated the occurrence of limited simplexin degradation by rumen microorganisms [22]. The bacterial isolates from these fermenter studies did not significantly decrease simplexin levels after incubation in selective media containing semi-purified simplexin. This suggests that the bacterial isolates obtained from the rumen-based in vitro fermentation were unable to degrade simplexin.

Previous studies have also indicated that rumen microbes are not able to degrade *Jatropha curcas* phorbol esters [23], although the presence of phorbol esters does impact on rumen microbial activities in a dose-dependent manner [24]. Phorbol esters present in *J. curcas* are structurally similar to simplexin but lack the epoxide and orthoester functionalities. Considerable success has been achieved in the detoxification of *Jatropha* seed cake for its use as animal feed by both physico-chemical and biological treatments [25]. *Pseudomonas* strains were reported to be particularly successful in detoxifying by hydrolysing the esters of phorbol esters [10,26,27]. Other bacteria and fungi have previously been reported to be capable of phorbol ester detoxification, including *Enterobacter* spp., *Bacillus* spp., and *Pleurotus* spp. [28,29,30]. It would be useful to look towards the microorganisms that have been found to successfully detoxify phorbol esters and examine whether they are similarly able to detoxify simplexin and produce any of the targeted H_2_SO_4_ hydrolysis products **8**, **9**, **10**, and **11**, although the relative stability of the simplexin orthoester [17] could be confounding.

### 2.5. UPLC-MS/MS Method for Simplexin Quantification and Simplexin Metabolite Identification in Lyophilised Cattle Blood

As part of this study, a further feeding trial was conducted with steers fed Pimelea plant material included in their daily diet for 11 weeks under animal ethics QAFFI/QASP/337/20/DAF, as reported in Fletcher et al. [22]. Steer jugular blood was collected weekly from the pre-treatment week to the final week with the anticipation that either simplexin or the targeted hydrolysis products **8**, **9**, **10**, and **11**, as shown in Figure 9, could be detected in these blood samples by the modified UPLC-MS/MS method described in Section 4.5. Simplexin analysis was conducted in an adaption of the previously described method [9], utilising dominant MS/MS transition to the major fragment ions for quantitation (*m*/*z* 533.3109 > 253.1223) and verification (*m*/*z* 533.3105 > 267.1380).

Preliminary analysis using dichloromethane:methanol (3:1) extraction and the initial UPLC-MS/MS method suggested simplexin was present in selected freeze-dried blood samples from cattle fed Pimelea. Further analysis of the blood samples showed that the result was a false positive. Troubleshooting was performed using the HRAM instrument, which revealed that phospholipids present in the blood may have been responsible for the interference. Therefore, a modified sample extraction with acetonitrile (ACN) was developed as described in Section 4.6 (iii). A modified UPLC-MS/MS method with LC elution (1% ACN) was also developed to monitor simplexin and formation of hydrolysis products **8**, **9**, **10**, and **11** using parameters described in Section 4.5. The modified methods were able to analyse simplexin in lyophilised blood with recoveries of 59% and 64% at 10 ng/g and 50 ng/g simplexin in freeze-dried blood spike concentration, respectively. The method also has a limit of detection (LOD) of 3 ng/g simplexin in freeze-dried blood and limit of quantitation (LOQ) of 9 ng/g simplexin in freeze-dried blood. Fletcher et al. [9] reported their LC-MS/MS method on an older triple quadrupole mass spectrometer had a LOD of 17 ng/g in freeze-dried blood. The current method on the Orbitrap showed simplexin LOD to be five times more sensitive when compared to the older triple quadrupole used in Fletcher et al. [9]. The high resolving power and mass accuracy of the Orbitrap [31,32] allowed simplexin analysis in very low concentrations and allows the identification of potential simplexin metabolites.

Even though some of the animals fed Pimelea developed significant signs of Pimelea poisoning, neither simplexin nor the hydrolysis products **8**, **9**, **10**, and **11** could be detected in any of the analysed blood samples. These results suggest both simplexin and the potential simplexin hydrolysis products, if present in blood, were below the LOD of the HRAM instrument. It is possible that these metabolites were formed and then metabolised further by rumen microbes, but this does not appear to be the major route of simplexin degradation. If rumen microbes were unable to hydrolyse simplexin to produce similar products then one could expect to detect simplexin by UPLC-MS/MS in circulating blood samples of animals fed Pimelea plant material, but this was not observed. 

The lipophilic simplexin is thought to behave in a similar manner to lipids inside the rumen where simplexin is released from ingested Pimelea by rumen microorganisms and is transported into the small intestine. In theory, simplexin would be absorbed into the intestinal epithelial cells and further transported by the lymphatic system, passing along the thoracic duct and entering the bloodstream near the heart where simplexin would be in contact with PKC enzymes. The absence of simplexin in circulatory blood suggests that this toxin may be strongly bound and removed by PKC enzymes such as those in pulmonary venules. In vitro assays with pulmonary vein smooth muscle suggest that this binding is strong (“virtually irreversible”) and the toxin difficult to “wash-out” [33,34]. Another possibility could be that the lipophilic simplexin could have been stored in lymph nodes within the lymphatic capillaries. Previous studies have reported that lymph nodes can store molecules before they are released back to the lymphoid fluid [35,36,37]. Therefore, it is possible that simplexin could be stored in lymph nodes and is slowly released into the bloodstream in low concentrations while remaining below the LOD of the Orbitrap.

## 3. Conclusions

Acid hydrolysis using both hydrochloric acid and sulfuric acid were shown to hydrolyse simplexin at both the epoxide and orthoester sidechains producing a series of products with HRAM data consistent with proposed structures. In this study, HRAM analysis proved invaluable in confirming, for the first time, the molecular composition of MS ion fragments derived from simplexin, which, by extrapolation, aided in the identification of hydrolysis products **5**, **6**, **7**, **8**, **9**, **10**, and **11** and also their MS fragmentation products. 

It was considered likely that simplexin could be hydrolysed by rumen microorganisms and form metabolites similar to the products observed from the sulfuric acid mediated hydrolysis. However, targeted UPLC-MS/MS analysis to search for such products in all fermentation fluid samples, bacterial isolate samples, and freeze-dried blood samples failed to detect these in either the in vitro or in vivo samples. This work was conducted without isolation of the putative hydrolysis products. Untargeted UPLC-MS/MS to search for metabolites associated with Pimelea poisoning was attempted but not successful due to the low levels of simplexin, which makes untargeted UPLC-MS/MS metabolite searching not feasible.

Further work could be performed on a larger scale to isolate and purify the hydrolysis products. Characterisation of the separated hydrolysis products using NMR would then help further confirm their structures. Isolated, purified, and characterised hydrolysis products could be used as future standards for their quantification using UPLC-MS/MS, and also enable the hydrolysis compounds to be tested for their toxicity. Blood samples can be used for further simplexin metabolite searches using targeted MS/MS analysis as there are other possible metabolite pathways that could detoxify simplexin, such as hydroxylation and/or conjugation [38,39,40,41,42].

## 4. Materials and Methods

### 4.1. Plant Material Collection and Processing

New growth and flowering tops of *Pimelea elongata* were collected from a property near Nebine, Queensland, in September 2017. Collection and global positioning system (GPS) coordinates were noted while plant identification was confirmed by Queensland Herbarium with a botanical specimen incorporated into their permanent collections as vouchers (AQ522516). Field-collected plant samples were air-dried in the laboratory. Roots of the plant were freed of soil, separated from the stalks and roots were milled using a Christy and Norris 8000RPM 8” Laboratory Mill (Ipswich, UK) fitted with a 3 mm screen. Milled root samples were kept frozen (−40 °C) until extraction for simplexin isolation.

### 4.2. Simplexin Isolation from Pimelea elongata Roots

Simplexin extraction and isolation from roots of *P. elongata* plant were based on the method previously reported by Chow et al. [1]. Milled root material (120.17 g) was soaked in 90% methanol (600 mL), sonicated for 30 min, and shaken overnight on a reciprocating shaker. The extract was filtered, and plant material rinsed with additional methanol (500 mL). The combined methanol extracts were concentrated under reduced pressure. The reduced extract was treated with saturated sodium chloride solution (10 mL) before extraction with dichloromethane (3 × 100 mL). The combined dichloromethane extracts were dried over anhydrous sodium sulfate (Na_2_SO_4_), filtered and concentrated under reduced pressure.

The residue was then partitioned between hexane (100 mL) and acetonitrile (3 × 50 mL). The combined acetonitrile layers were washed with hexane (50 mL) and concentrated under vacuum. Residue (0.7 g) was subjected to TLC visualisation on silica gel 60 F_254_ (Merck, Castle Hill, NSW, Australia) eluted with ethyl acetate/hexane, at a 1:1 ratio. Visualisation was conducted by ultraviolet light at 254 nm and by a reagent consisting of ammonium metavanadate (4 g) dissolved in 50% sulphuric acid (200 mL) prepared based on method described in Sakata et al. [17]. Simplexin (**1**) gave a non-distinctive dark grey colour on TLC plates under UV light while simplexin dipped in ammonium metavanadate solution gave red-brown spot on standing (R_f_ 0.2). The residue was dissolved in dichloromethane, preadsorbed on silica (2 g), and subjected to flash chromatography (diameter 20 mm, length 370 mm, 10 cm silica, hexane then 5% ethyl acetate/hexane up to 100% ethyl acetate), producing 11 fractions. Fractions containing simplexin, eluting with 35–45% ethyl acetate/hexane, were identified using TLC and confirmed by the UPLC-MS/MS method described in Section 4.5. 

Fractions containing simplexin (Fractions 6–10) were combined and then concentrated under reduced pressure to give a residue (179 mg), which was preadsorbed onto 1 g silica (from dichloromethane) and subjected to flash chromatography (diameter 20 mm, length 440 mm, 8 g silica, 25% ethyl acetate/hexane) for further purification. Fractions 1–12 were collected using 25% ethyl acetate/hexane while fractions 13–41 were collected using 35% ethyl acetate/hexane. Fractions containing simplexin were identified using TLC and confirmed by UPLC-MS/MS analysis as described in Section 4.5. Fractions with simplexin (Fractions 21–35) were combined and concentrated under reduced pressure to give crude simplexin (66.6 mg).

The residue was purified using preparative HPLC via a Shimadzu Class-VP HPLC system consisting of an auto-injector (Shimadzu SCL-10A VP), column oven and UV-Vis detector controlled by Shimadzu Class-VP software (Shimadzu, Kyoto, Japan). Purification was conducted using Phenomenex Luna 5µ C_18_(2) (250 mm × 15 mm, 5 µm) column at a flow rate of 8 mL/min with column temperature set at 40 °C. Mobile phase used was water (A) and methanol (B) with isocratic elution set to 95% B for 15 min. Injection volume was 500 µL with residue concentration of 10 mg/mL methanol per injection. UV-Vis detector was set at wavelengths 232 nm and 242 nm. Eluted simplexin was collected into one fraction. Simplexin was confirmed using the UPLC-MS/MS method, as described in Section 4.5. Combined fractions were concentrated under reduced pressure and freeze-dried (CSK Climatek, Darra, Australia) overnight to produce solid yellow crystals of purified simplexin (24.4 mg, 0.02% yield, >95% purity, confirmed by NMR and LC-MS analyses). Purified simplexin (**1**) was used as the starting material for the acid mediated hydrolysis and as a calibration standard during UPLC-MS/MS analysis.

### 4.3. Hydrochloric Acid Hydrolysis of Simplexin to Give Products ***5***, ***6*** and ***7***

The hydrolysis method was based on a cyclic orthoacetate hydrolysis reported by Wang et al. [43] with some modifications. Simplexin (**1**) (1 mg) was dissolved in tetrahydrofuran (4 mL) and then aqueous 6 N HCl (2 mL) was added. The mixture was stirred for five days at ambient laboratory temperature (22 °C). TLC on silica gel 60 F_254_ (ethyl acetate/hexane, 1:1) suggested complete conversion of simplexin (R_f_ 0.3) to a lower R_f_ product (R_f_ 0.0). The mixture was diluted with water (5 mL) and extracted with ethyl acetate (3 × 15 mL). The combined extracts were dried with anhydrous sodium sulfate (Na_2_SO_4_) and concentrated under vacuum to give a crude product mixture that was dissolved in methanol, was filtered via 0.2 µm membrane syringe filters (GHP Acrodisc, Pall, Cheltenham, Australia), and analysed by UPLC-MS/MS to show the presence of the hydrolysis products **5**, **6**, and **7**, as described in Section 4.5.

### 4.4. Sulfuric Acid Hydrolysis of Simplexin to Give Products ***8***, ***9***, ***10*** and ***11***

The hydrolysis procedure was based on Wang et al. [43] with modifications. Simplexin (**1**) (10 mg) was dissolved in tetrahydrofuran (4 mL) and aqueous 6 N H_2_SO_4_ (2 mL). The mixture was heated for two days at 60 °C. TLC on silica gel 60 F_254_ (ethyl acetate/hexane, 1:1) showed complete conversion of simplexin (R_f_ 0.2) to a lower R_f_ product (R_f_ 0.0). The mixture was diluted with water (5 mL) and extracted with ethyl acetate (3 × 20 mL). The combined extracts were dried over anhydrous Na_2_SO_4_ and reduced under vacuum to give a crude mixture that was dissolved in methanol, filtered via 0.2 µm membrane syringe filters (GHP Acrodisc, Pall, Cheltenham, Australia), and analysed by UPLC-MS/MS analysis to show the presence of the hydrolysis products **8**, **9**, **10**, and **11**, as described in Section 4.5.

### 4.5. Analysis of Simplexin and Simplexin Hydrolysed Products by UPLC-MS/MS

Sample separation was performed on Thermo Fisher Scientific Ultimate 3000 Rapid Separation (RS) UPLC system (Thermo Fisher Scientific, Waltham, MA, USA) equipped with an autosampler, a RS pump, and a temperature control column compartment. Waters Acquity UPLC BEH Shield RP18 column (2.1 × 100 mm, 1.7 µm) was used with column temperature set to 35 °C with flow rate of 0.3 mL/min. Mobile phase used was H_2_O + 10 mM ammonium formate + 0.1% formic acid (A) and methanol + 10 mM ammonium formate + 0.1% formic acid (B) with the following gradient: 0 min, 5% B; 0.3 min, 5% B; 0.5 min, 80% B; 0.8 min, 93% B; 1.3 min, 99% B; 5.8 min, 99% B; 6 min, 5% B; 7 min, 5% B. Injection volume was set to 6 µL for simplexin hydrolysis with HCl while 10 µL was used for simplexin hydrolysis with H_2_SO_4_.

MS parameters and inclusion list were tuned for simplexin and hypothetical HCl and H_2_SO_4_ hydrolysis products, respectively. MS detection was performed using Thermo Scientific Q-Exactive Orbitrap HRAM spectrometer (Thermo Fisher Scientific, Waltham, MA, USA) equipped with heated electrospray ionisation (HESI) probe used in positive ionisation mode. Orbitrap MS system was tuned and calibrated in both positive and negative ionisation modes using infusion of standard mixtures. MS/MS was operated in PRM mode with inclusion list tailored for simplexin. UPLC-MS/MS HRAM system was controlled using Xcalibur 4.1 software (Thermo Fisher Scientific, Waltham, MA, USA). HESI probe in positive ionisation mode was optimised for simplexin; sheath gas flow rate 45 arbitrary units, auxiliary gas flow rate 10 arbitrary units, sweep gas flow rate 2 arbitrary units, spray voltage 3.5 kV, spray current 5 µA, capillary temperature 250 ℃, S-lens RF level 50 arbitrary units and auxiliary gas heater temperature 400 ℃. Full MS parameters were shown as follows; Full MS-SIM parameters were set with resolution setting of 70,000 (R_FWHM_ at *m*/*z* 200), full MS mass range of *m*/*z* 80–1200, automatic gain control target of 3 × 10^6^ and maximum injection time of 200 ms. MS/MS (PRM) parameters were set to resolution 70,000 (R_FWHM_ at *m*/*z* 200) with automatic gain control target of 2 × 10^5^, maximum injection time of 100 ms, isolation window of *m*/*z* 4 and loop count set to 1. The inclusion list and normalised collision energy used for both HCl and H_2_SO_4_ hydrolysis products are shown in Table 2. MS/MS fragments of simplexin and simplexin hydrolysed products was identified using Thermo Xcalibur Qual Browser version 4.0.27.19 (Thermo Fisher Scientific, Waltham, MA, USA).

A modified UPLC-MS/MS method was used for the analysis of freeze-dried cattle blood samples for simplexin and simplexin metabolites. Sample separation was performed using the same UPLC system, column and MS/MS conditions as described above. The mobile phase was modified to H_2_O + 10 mM ammonium formate + 0.1% formic acid (A), methanol + 10 mM ammonium formate + 0.1% formic acid (B) and acetonitrile (C) with the following gradient: 0 min, 5% B, 1% C; 0.3 min, 5% B, 1% C; 0.5 min, 80% B, 1% C; 0.8 min, 93% B, 1% C; 1.3 min, 98% B, 1% C; 5.8 min, 98% B, 1% C; 6 min, 99% B; 8.8 min, 99% B; 9 min, 5% B, 1% C; 11 min, 5% B, 1% C. Injection volume was set to 10 µL.

### 4.6. Analysis of Biological Samples for Acid Hydrolysed (H_2_SO_4_) Simplexin Products

Daily fermentation fluid samples and fermentation fluid samples from simplexin degradation assays, bacterial isolate samples from isolate simplexin degradation assays and freeze-dried blood samples of Pimelea affected cattle from animal trial were analysed for simplexin hydrolysis products **8**, **9**, **10** and **11** using the UPLC-MS/MS method targeting H_2_SO_4_ hydrolysis products described above. The samples were analysed using UPLC-MS/MS method described in Section 4.5.

(i) Daily fermentation fluid samples and simplexin degradation assay samples were collected from rumen-based in vitro fermentation trials as reported in Fletcher et al. [22]. In vitro fermentations were performed for a set duration of time using a benchtop fermentation system (Labfors 3, Infors HT, Bottmingen, Switzerland) containing milled Pimelea plant and rumen fluid base starter with rumen fluid collected from Pimelea resistant animals under animal ethics committee (AEC) approvals SA 2016/11/586 Department of Agriculture and Fisheries AEC, November 2016; SA 2019/11/722 Department of Agriculture and Fisheries AEC, August 2019; SAFS/296/17 The University of Queensland AEC, July 2017. Fermentation fluid was removed daily for sampling and was replaced with fermenter salts solution containing salts and minimal nutrients. Selected fermentation fluid day samples were used for simplexin degradation assays, incubated for 168 h and sampled at predetermined time interval for simplexin analysis. Both fermentation fluid samples were extracted using methanol followed by solid phase extraction (SPE) and filtered as reported in Fletcher et al. [22] prior to UPLC-MS/MS analysis.

(ii) Bacterial isolate samples were sampled from isolate simplexin degradation assays as reported in Fletcher et al. [22]. Simplexin degradation assays were performed using multiple selective media containing simplexin to screen selected rumen bacterial isolates for their ability to degrade simplexin. Negative controls and isolate cultures were sampled at time 0 h prior to incubation. Both negative control and isolate cultures were incubated at 39 °C for 168 h. Samples were extracted with methanol followed by SPE clean-up and filtered as reported in Fletcher et al. [22] for UPLC-MS/MS analysis.

(iii) Blood samples were collected from a pen trial (QAFFI/QASP/337/20/DAF The University of Queensland AEC, September 2020) in which 40 steers were fed with Pimelea plant material as part of their daily diet for 11 weeks as reported in Fletcher et al. [22]. Steer jugular blood was collected weekly from the pre-treatment week to the final week of the trial into Vacutainer tubes containing lithium heparin anticoagulant (2 × 10 mL, Becton Dickinson, Franklin Lakes, NJ, USA). Both lithium heparin blood tubes were combined into a 50 mL centrifuge tube, stored frozen (−20 °C) and freeze-dried. Freeze-dried blood samples (0.5 g) were extracted by acetonitrile (15 mL) and the solvent was removed under nitrogen flow. The residue was redissolved in dichloromethane (15 mL) and washed with saturated sodium chloride solution (6 mL). The organic layer was dried with anhydrous sodium sulfate and evaporated under nitrogen flow. The residue was partitioned between hexane (4 mL) and acetonitrile (4 mL) with the acetonitrile layer collected. Acetonitrile layer was evaporated under nitrogen flow and redissolved with methanol (0.5 mL). Extracted freeze-dried blood samples were filtered prior to UPLC-MS/MS analysis using the modified UPLC-MS/MS method described in Section 4.5.

## Figures and Tables

**Table 1:** HRAM data for simplexin (**1**) and its percentage relative intensity of selected fragmentation ions in positive ionisation mode.

Molecular Formula (M)	RT	Adduct (Calculated *m*/*z*)	Selected Fragmentation Ions (% Relative Intensity)
C_30_H_44_O_8_	5.43	[M + H]^+^ *m*/*z* 533.3109	343.1543 (2), 325.1433 (4), 307.1333 (7), 297.1492 (7), 279.1378 (17), 267.1381 (62), 253.1220 (100)

**Table 2:** Inclusion list and normalised collision energy in positive ionisation mode for simplexin (**1**) and proposed hydrolysis products **5**, **6**, **7**, **8**, **9**, **10**, and **11**, as depicted in Figure 6 and Figure 9.

Hydrolysis Method	Molecular Formula (M)	Species	Calculated Molecular Ion (*m*/*z*) [M + H]^+^ *	Normalised Collision Energy (eV)	Proposed Product and Structure Number
HCl	C_30_H_44_O_8_	+ H^+^	533.3109	30	simplexin (**1**)
	C_30_H_45_ClO_8_	+ H^+^	569.2876	30	chlorohydrin (**5**)
	C_20_H_29_ClO_8_	+ H^+^	433.1624	30	chloropolyol (**6**)
	C_30_H_47_ClO_9_	+ H^+^	587.2981	30	monoester chlorohydrin (**7**)
H_2_SO_4_	C_30_H_44_O_8_	+ H^+^	533.3109	35	simplexin (**1**)
	C_20_H_24_O_6_	+ H^+^	361.1646	35	polyol (**8**)
	C_30_H_42_O_7_	+ H^+^	515.3003	35	monoester (**9**)
	C_30_H_46_O_9_	+ H^+^	551.3215	35	pentol (**10**)
	C_30_H_48_O_10_	+ H^+^	569.3320	35	monoester polyol (**11**)

* Calculated [M + H]^+^ from molecular formula was based on isotope ^35^Cl.

## Data Availability

The data supporting this study is provided within this article and its Supplementary Information.

## Supplementary Materials

The following supporting information can be downloaded at: https://www.mdpi.com/article/10.3390/toxins15090551/s1, Figure S1: Molecular ions of chlorohydrin (**5**), chloropolyol (**6**) and monoester chlorohydrin (**7**) in positive ionisation mode showing distinctive isotope patterns due to relative abundances of ^35^Cl and ^37^Cl of [M + H]^+^ peaks [C_30_H_45_ClO_8_ + H]^+^, [C_20_H_30_ClO_8_ + H]^+^ and [C_30_H_47_ClO_9_ + H]^+^ respectively. (i) Observed hydrolysis product molecular ion isotope pattern and (ii) calculated molecular ion isotope pattern.
