# Supplementary material for: Identification of Acid Hydrolysis Metabolites of the Pimelea Toxin Simplexin for Targeted UPLC-MS/MS Analysis
Source: Toxins (Basel). 2023 Sep 5;15(9):551. doi: 10.3390/toxins15090551 (PMC10535249; doi:10.3390/toxins15090551)
Supplement: Supplementary file 1 [file toxins-15-00551-s001.zip › toxins-2526349-supplementary.pdf]

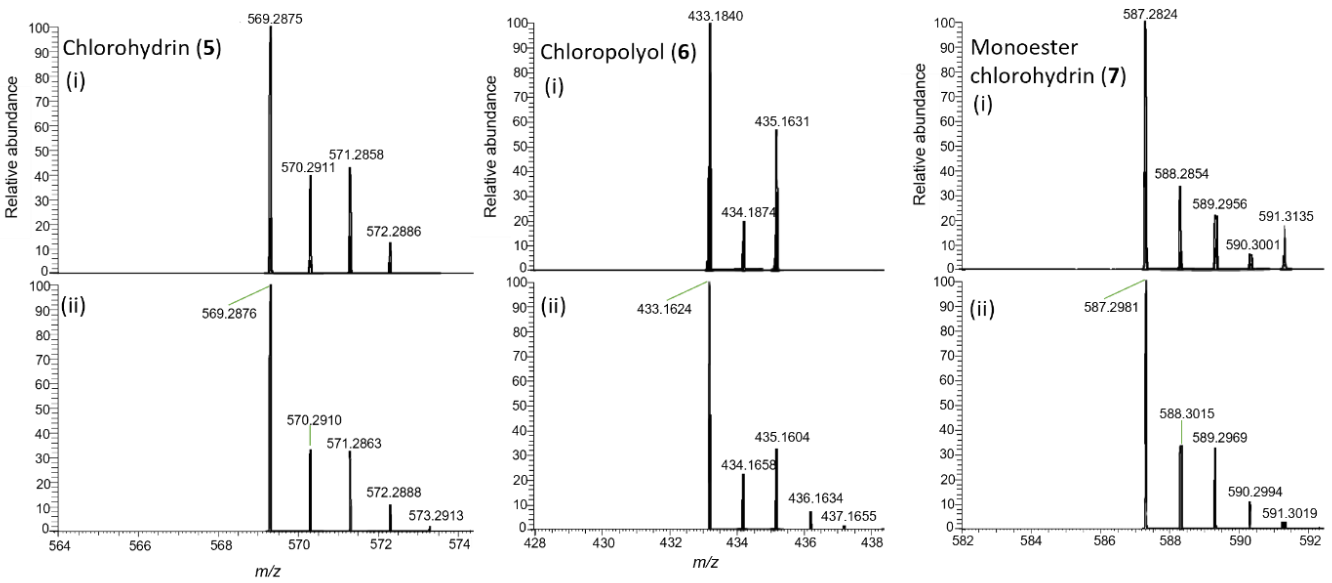

Figure S1. Molecular ions of chlorohydrin (**5**), chloropolyol (**6**) and monoester chlorohydrin (**7**) in positive ionisation mode showing distinctive isotope patterns due to relative abundances of ^{35}Cl and ^{37}Cl of $[M + H]^+$ peaks $[C_{30}H_{45}ClO_8 + H]^+$, $[C_{20}H_{30}ClO_8 + H]^+$ and $[C_{30}H_{47}ClO_9 + H]^+$ respectively. (i) Observed hydrolysis product molecular ion isotope pattern and (ii) calculated molecular ion isotope pattern.
